# Fostering trust in digital respiratory medicine: the open access ERS CONNECT digital health technology repository

**DOI:** 10.1183/13993003.00448-2026

**Published:** 2026-07-02

**Authors:** Isaac Cano, Amy Hai Yan Chan, Richard W. Costello, Kjeld Hansen, Mark Hew, Vitalii Poberezhets, Hilary Pinnock, Tamara Vagg, Job F.M. van Boven

**Affiliations:** 1 Hospital Clínic de Barcelona, Clinical Informatics Service, Fundació de Recerca Clinic Barcelona - Institut d'Investigacions Biomèdiques August Pi i Sunyer (FRCB-IDIBAPS), Universitat de Barcelona, Barcelona, Spain; 2 School of Pharmacy, Faculty of Medical and Health Sciences, The University of Auckland, Auckland, New Zealand; 3 Department of Respiratory Medicine, RCSI University of Medicine and Health Sciences, Dublin, Ireland; 4 Department of Respiratory Medicine, Beaumont Hospital, Dublin, Ireland; 5 School of Innovation, Economics and Technology, Kristiania University of Applied Sciences, Oslo, Norway; 6 European Lung Foundation, Sheffield, UK; 7 Asthma, Allergy and Clinical Immunology, Respiratory Medicine, Alfred Hospital, Melbourne, Australia; 8 Public Health and Preventive Medicine, Monash University, Melbourne, Australia; 9 Department of Propedeutics of Internal Medicine, National Pirogov Memorial Medical University, Vinnytsya, Ukraine; 10 The University of Edinburgh, Usher Institute, Edinburgh, UK; 11 Cork Centre for Cystic Fibrosis (3CF), Cork University Hospital, University College Cork, Cork, Ireland; 12 Department of Clinical Pharmacy and Pharmacology, Groningen Research Institute for Asthma and COPD (GRIAC), University Medical Center Groningen, University of Groningen, Groningen, The Netherlands

## Abstract

Reflecting broader societal trends, respiratory medicine is undergoing a profound transformation, driven by an exponential increase in the availability of digital health technologies [1, 2]. These innovations range from wearable biomarker sensors and digital “smart” inhalers to artificial intelligence (AI)-driven diagnostic algorithms and use of generative AI to support clinician–patient communication. Digital health technologies hold substantial promise for advancing personalised care and accelerating respiratory research; however, this rapid proliferation has created a paradoxical challenge.

## Shareable abstract

**Access to digital health tools is rapidly expanding, but adoption in respiratory care remains fragmented. The ERS CONNECT Repository provides a trusted, evidence-based resource to bridge innovation and practice and support meaningful implementation.** https://bit.ly/3Pn2prp

*To the Editor:*

Reflecting broader societal trends, respiratory medicine is undergoing a profound transformation, driven by an exponential increase in the availability of digital health technologies [1, 2]. These innovations range from wearable biomarker sensors and digital “smart” inhalers to artificial intelligence (AI)-driven diagnostic algorithms and use of generative AI to support clinician–patient communication. Digital health technologies hold substantial promise for advancing personalised care and accelerating respiratory research; however, this rapid proliferation has created a paradoxical challenge. While the quantity of available tools has skyrocketed, their successful implementation into routine clinical practice remains sporadic and fragmented. There is a critical gap between technological innovation, its strategic application and real-world implementation.

Respiratory clinicians today face a “digital jungle”. Navigating thousands of available health applications to find those that are clinically validated, regulatory-compliant, accessible, suitable, safe and meaningful for patients can be daunting. The lack of standardised evaluation frameworks has led to “choice paralysis”, where effective solutions are lost amidst a sea of unregulated wellness products. Furthermore, the lack of interoperability with existing digital platforms and absence of clear reimbursement pathways has stifled the scalability of even the most promising innovations [3].

Europe is responding to these systemic challenges with high-level regulatory harmonisation, most notably through the proposed European Health Data Space [4]. The European Health Data Space aims to create a trustworthy framework for the primary and secondary use of health data across borders, fostering a single market for digital health services. Although these initiatives primarily govern health data standards rather than individual technologies, they directly influence how data generated by patient-facing respiratory tools can best be integrated into a clinical workflow. Echoing a key message of a report of the World Health Organization (WHO) Regional Office for Europe and the European Respiratory Society (ERS) [5], there is a need for practical mechanisms that bring technology owners, clinicians and users together to ensure that digital innovation is not only compliant, but also clinically meaningful and implementable [6].

Aligned with the ERS digital strategy [7], the Clinical Research Collaboration “CONNECT” has recently launched a digital respiratory technology repository [8]. Developed in collaboration with the European Union Digital Health Uptake (DHU) initiative, this repository represents an important step towards operationalising trust in digital respiratory medicine.

The CONNECT repository (figure 1) has established a rigorous evaluation framework co-designed by a multidisciplinary panel. Technologies’ credentials are assessed against four critical pillars: clinical evidence, regulatory compliance (including the Medical Device Regulation and the General Data Protection Regulation), usability, and implementation feasibility. This vetting process addresses the primary barrier to adoption: uncertainty regarding trust. Technologies meeting these criteria receive a “CONNECT Listed” distinction, which serves as a visual marker that the technology has achieved appropriate regulatory status for a medical device. The repository also summarises evidence and provides links to enable potential users to better understand the technology.

**FIGURE 1:**
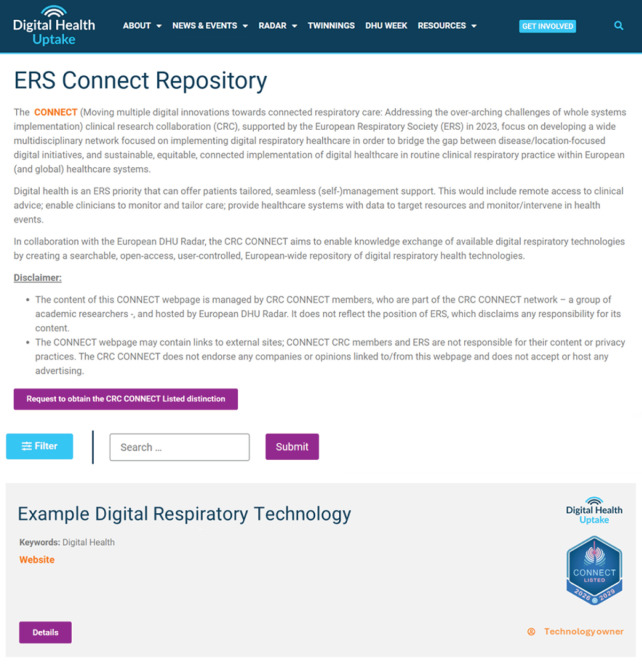
Landing page of the European Respiratory Society (ERS) CONNECT repository with an example listed digital respiratory technology. The ERS CONNECT repository is hosted within the European Union Digital Health Uptake (DHU) Radar repository. Only respiratory technologies that satisfy criteria for clinical evidence, regulatory compliance, usability, and implementation feasibility receive the “CONNECT Listed” designation and are listed at https://digitalhealthuptake.eu/ers-connect-repository.

Trust in technology underpins relationships in digital care [9]. The relevance of this repository extends beyond simple listing and compilation; it acts as a freely accessible, centralised source of verifiable knowledge for the respiratory community. By filtering for quality and compliance, the repository reduces the due diligence burden on individual healthcare providers and can reassure patients and users. It facilitates an efficient “matchmaking” process, where clinicians can identify tools that fit their specific patient pathways, and technology developers can showcase their adherence to rigorous standards.

This initiative aligns with broader international efforts to advance digital health implementation, including the WHO's Global Strategy on Digital Health 2020–2025 [10] and the Digital Health Atlas. While directly supporting European Health Data Space goals, the ERS CONNECT repository's evaluation framework reflects principles applicable worldwide. The ERS strategic aim to support ethical, safe and equitable use of digital health technology resonates with global efforts to ensure technologies serve populations across diverse healthcare contexts.

The success of this initiative relies on community engagement of diverse stakeholders ranging from clinicians to patients, to the public and to industry. The repository is now active and hosted by the DHU Radar, ready to serve as a bridge between innovation and practice. We invite technology developers – from academic spin-offs to established industry leaders – to submit, by means of a short online questionnaire, their respiratory solutions for review. Simultaneously, we encourage the CONNECT network of currently around 1000 clinicians and researchers to suggest trusted technologies for the repository and use this resource to inform their practice.

By consolidating ERS efforts through platforms such as the ERS CONNECT repository, we can move beyond the current fragmentation to a more streamlined, cohesive system. We can ensure that the digital future of respiratory medicine is defined not only by the volume of technology available, but by the trustworthiness, quality and clinical impact of the digital solutions available for implementation.
